# Computational Evaluation of the Use of Fluorescein Isothiocyanate as a Preliminary Test for Amphetamines and Cathinones

**DOI:** 10.1021/acsomega.5c04896

**Published:** 2025-08-12

**Authors:** Caio Henrique Pinke Rodrigues, Aline Thais Bruni

**Affiliations:** Departmento de Química, Faculdade de Filosofia, Ciências e Letras de Ribeirão Preto, 124588 Universidade de São Paulo. Avenida Bandeirantes, 3900 - CEP, 14040-901 Ribeirão Preto, SP, Brazil

## Abstract

The rise of new psychoactive substances (NPS) challenges traditional drug detection, especially in initial analyses. This research investigates fluorescein isothiocyanate (FITC) as a potential colorimetric reagent for detecting amphetamines and related cathinones through computational and mechanistic methods. Forty-two amphetamine-type stimulants were examined using density functional theory (DFT) and UV–vis spectra via TD-DFT. Results showed FITC interacts specifically with primary and secondary amines, causing lower excitation energies and spectral shifts into the visible spectrum. However, statistical tests—Pearson’s correlation, PCA, and ANOVA—indicate that this chromophore has limited ability to distinguish between amphetamines and cathinones. Hence, FITC-based tests might be useful as part of a series of presumptive assays but are not suitable as a sole method. Furthermore, the study highlights how in silico methods can effectively evaluate new reagents for forensic purposes, providing faster and more cost-efficient alternatives to experimental testing.

## Graphical abstract

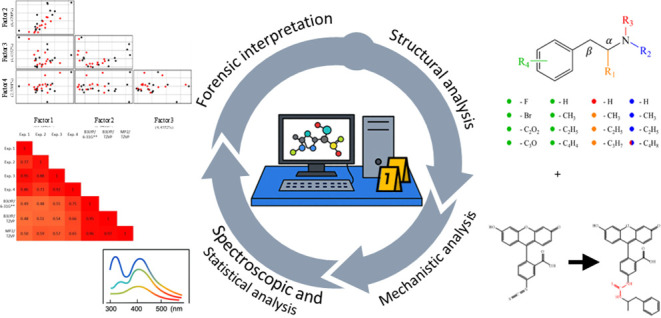

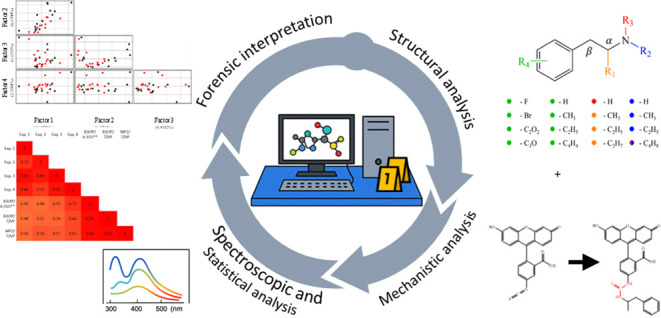

## Introduction

The phenomenon known as New Psychoactive Substances (NPS)^1–3^ gained worldwide prominence in the early 2000s. These substances have been developed to mimic the effects of classic drugs, making the identification process more challenging, among several other purposes. Over these decades, one of the most prominent groups of NPS was the amphetamine class and its structural analogues, known as cathinones.^3–7^

The Scientific Working Group (SWGDRUG) recommends forensic techniques for analyzing seized drugs, setting minimum standards for identification, categorized into A, B, and C based on their discriminatory ability.^8,9^ Techniques with general or class-based selectivity, like colorimetric tests and UV–vis, are Category C, mainly used as preliminary on-site tests. While colorimetric tests are sensitive to functional groups, they lack specificity, serving primarily as screening tools for drug presence or absence.^10^

Among the new possibilities are compounds that absorb and emit light in the ultraviolet–visible spectrum range, such as quinines,^11,12^ rhodamines,^13^ bodipy,^14^ calixarenes,^15^ anthracenes^16^ and fluoresceins.^17^ However, due to the vast structural possibilities, more traditional tests can result in false negatives,^18^ the Simon Test is an example, as it indicates the presence of primary and secondary amines, but not tertiary amines.^19^ One way to avoid these negative outcomes, besides proposing new structures, is to modify existing compounds structurally. For example, fluorescein can be functionalized with the isothiocyanate group (N=C=S) gives rise to fluorescein isothiocyanate (FITC)^17,20^ and this change enables the reaction with compounds that have primary and secondary amines.^20^ These luminescent probes have been studied using in silico methodologies, that is, by computational means, to obtain faster responses to the complexities and challenges of drugs.

Given the above, this study aims to evaluate, through different approaches, the use of FITC as a more specific and less costly preliminary test for the detection of NPS, specifically amphetamines and cathinones. The study investigated the structures of amphetamine and FITC by conformational analysis using density functional theory. The results served as the basis for a reaction analysis and spectral analysis in the ultraviolet–visible region, with all steps supported by univariate and multivariate statistical analyses.

## Experimental Section

### Study System

The structures of interest represent 21 molecules of the ATS (Amphetamine-type stimulants) type.^21^ These molecules were represented according to the substituents in Figure 1,^22^ but the complete structures can be analyzed in the Supporting Information. The abbreviation used for amphetamines was *a*_*n*_, where *n* varied from 1 to 21.

**Figure 1:**
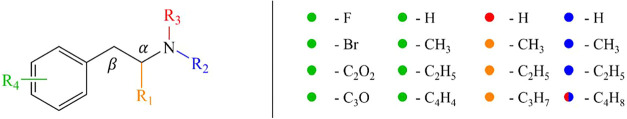
Schematic representation of the symbols employed in the Study system.

### Computational Procedure

In this study, simulations were performed to (i) optimize the individual structures and those complexed with FITC; and (ii) calculate the excitation of both previous results. After obtaining these data, a statistical evaluation was performed. In more detail, this work was divided into three parts, according to the objectives:

(a) STEP I—structural and electronic analysis of amphetamine to ensure the lowest energy conformation;
(b) STEP II—in this step of the work, absorption spectra in the ultraviolet–visible region were obtained for all structures (individual and after the thiourea bond) with the respective evaluations; and
(c) STEP III—finally, the statistical distinction between the spectra of those amphetamines and cathinones was verified.

### STEP I—Structural and Electronic Assessment

Conformational analysis is an essential step for verifying possible structures when crystallographic data are lacking or divergent.^23^ According to the International Union of Pure and Applied Chemistry (IUPAC), conformational analysis is “the evaluation of the relative energies (or thermodynamic stabilities), reactivities, and physical properties of alternative conformations of a molecular entity”. There are methods suitable for this purpose. In this work, a systematic analysis was used. This approach explores the conformational space through discrete variations of the dihedral^24^ that will compose the potential energy surface (PES—Potential Energy Surface). The number of possible conformations is given by eq 1, where the dihedral increment of angle *i* (θ_*i*_) is used to determine the number of conformers (S) relative to the number of free rotation angles (*N*).^25^

1 $$S={\left(\frac{360}{\theta_{i}}\right)}^{N}$$

One way to assess the quality of conformational analysis is to compare the obtained molecular geometries with experimental data.^26–28^ This practice uses coordinates taken from X-ray structures and compares them with those optimized by computational methods, and the agreement between them is verified.^29^

The systematic conformational analysis was performed using the Orca v4.1.0 software with the Scan key term at a DFT (Density Functional Theory) B3LYP^30,31^ level of theory with TZVP basis^32^ and D3BJ correction.^33–36^ The conformers were obtained by rotating them 5° at a rate of 5°, and 72 conformers were obtained. After the calculation, the energies of each conformer were used to perform the Boltzmann Distribution (*p*_*i*_). This approach evaluates the probability that the conformations (*i*) – obtained computationally – occur in solution at a given temperature (*T*). The diversity of conformers in equilibrium at 298.15 K can be measured by eq 2

2 $$p_{i}=\frac{\exp\left(-\frac{E_{i}}{k_{B}T}\right)}{\sum_{j=1}^{N}\;\exp\left(-\frac{E_{i}}{k_{B}T}\right)}$$

where *k*_B_ is the Boltzmann constant (0,001987 kcal mol^–1^ K^–1^), *N* is the number of conformers, and *E*_*i*_ is the electronic energy of conformer *i* in the ground state.^37,38^ All selected values have been normalized.

With the lowest energy structure defined, the variations in bond length and angle, as well as the dihedral angle values of the computationally obtained structures (with B3LYP/TZVP – in this study – and with B3LYP/6–31G** - in previous studies^22^) were evaluated. In addition, only this same structure was optimized with MP2 (second-order Møller–Plesset)^39–41^ based on TZVP to be used as a computational reference. The experimental structures’ data were obtained from the Cambridge Crystallographic Data Centre (https://www.ccdc.cam.ac.uk/).

All data from these structures were obtained using the Avogadro,^42^ software and analyzed using the Pearson correlation coefficient (r) .^43,44^ We were calculating the similarities between the bond lengths and angles of the experimental and simulated structures. The Pearson correlation is by eqs 3 [Disp-formula eq4],^43,44^ respectively:

3 $$r=\frac{\sum_{i}\left(x_{i}-\mathrm{x̅}\right)\cdot \left(y_{i}-\mathrm{y̅}\right)}{\sqrt{\sum_{i}{\left(x_{i}-\mathrm{x̅}\right)}^{2}\cdot \sum_{i}{\left(y_{i}-\mathrm{y̅}\right)}^{2}}}$$

4 $$\mathrm{bias}=\frac{\sum \left(y_{i}-\mathrm{y̅}\right)}{N}$$

Where *x̅* and *y̅* are the mean values for the structural parameters, and *x_i_* and *y_i_* are the *i*th structural values of the molecules evaluated. For Pearson’s correlation, this coefficient allows for quantifying the strength of the two variables. If *r* = 1, the relationship is positive and the variables are directly proportional. If *r* = −1, the association indicates that the variables are inversely proportional. The responses obtained through this analysis were analyzed to compose a heat map.^43^ These analyses were performed in an electronic spreadsheet (Excel, Microsoft Office 365).

### STEP II—Spectroscopic Evaluation

After the analysis of STEP I, with the optimized structures, the reaction products (Figure 2) for the 14 viable amphetamines with fluorescein isothiocyanate (FITC) were constructed. The Avogadro software was used for this purpose.^42^

**Figure 2:**
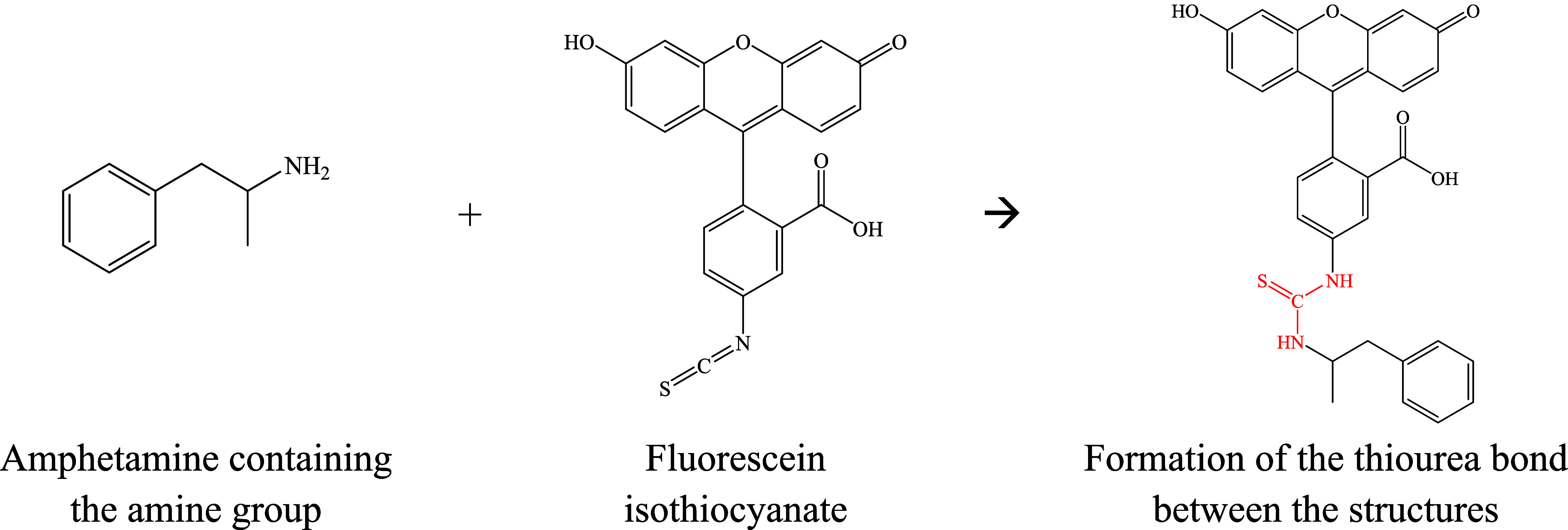
Representation of the reaction between amphetamine (a01) and fluorescein isothiocyanate for the formation of the thiourea bond (in red) and, thus, the product.

These structures were optimized at the DFT level with the ORCA v4.1.0 software,^45,46^ using the B3LYP functional^30,31^ with the 6–31G** basis,^47^ due to previous work with cathinones. All calculations indicated real frequencies (positive eigenvalues), ensuring the structures had a minimum energy conformation. These structures served as input for an energy calculation (single point) using the TD-DFT (Time-Dependent Density Functional Theory) approach.^48–50^ This calculation makes it possible to obtain data on the excited state and thus obtain the spectrum in the ultraviolet–visible (UV–vis) region.^45,46^ In addition to using the same level of optimization theory, an approximation for the Coulomb energy was used using RIJCOSX^51^ and a correction factor (scaling factor) to adjust the deviations of the theoretical results compared to the experimental responses.^52^ For this case study, the value used for correction was 0.99.^53^ These responses were inserted into a Gaussian distribution to ensure the data set had the same dimension. The resolution was 5 nm. The wavelength range varied from (i) 400 to 100 nm for the initial molecules; and (ii) 600 to 400 nm for the reaction product. The generated data were organized in a spreadsheet (Excel, Microsoft Office 365).

### STEP III—Statistical Evaluation of Amphetamines and Cathinones

To conduct this step, the data were organized into a matrix with 51 variables (wavelengths) and 28 samples (14 amphetamines and 14 cathinones linked to FITC). Due to the number of variables (dimensions), Principal Component Analysis (PCA)^54^ was the multivariate technique chosen to analyze these data. This technique reduces the system’s dimension to better understand the data, without losing information.^55^ In addition, it will enable the evaluation of the characteristics and natural similarities of the clusters.^56^ Since the data are from the same source, no pretreatment was applied to the DFT calculations. The analysis was conducted using the Pirouette v.4.5 software (Infometrix).

Finally, the ANOVA (analysis of variance) statistical method was used to determine whether the means of all computationally obtained spectra—across all structures and solvents—are equal. For this analysis, two hypotheses were considered: (i) the null hypothesis (*H*_0_:μ_1_ = μ_2_ = ... = μ_*k*_), stating that all means are equal; and (ii) the alternative hypothesis (*H*_1_ or *H*_α_), suggesting that at least one mean differs. ANOVA employs the F statistic (F ratio) to compare variability between groups with variability within groups.^57^ These analyses were conducted using spreadsheets (Excel, Microsoft Office 365).

Figure 3 presents a schematic diagram that summarizes the methodological workflow employed in this study.

**Figure 3:**
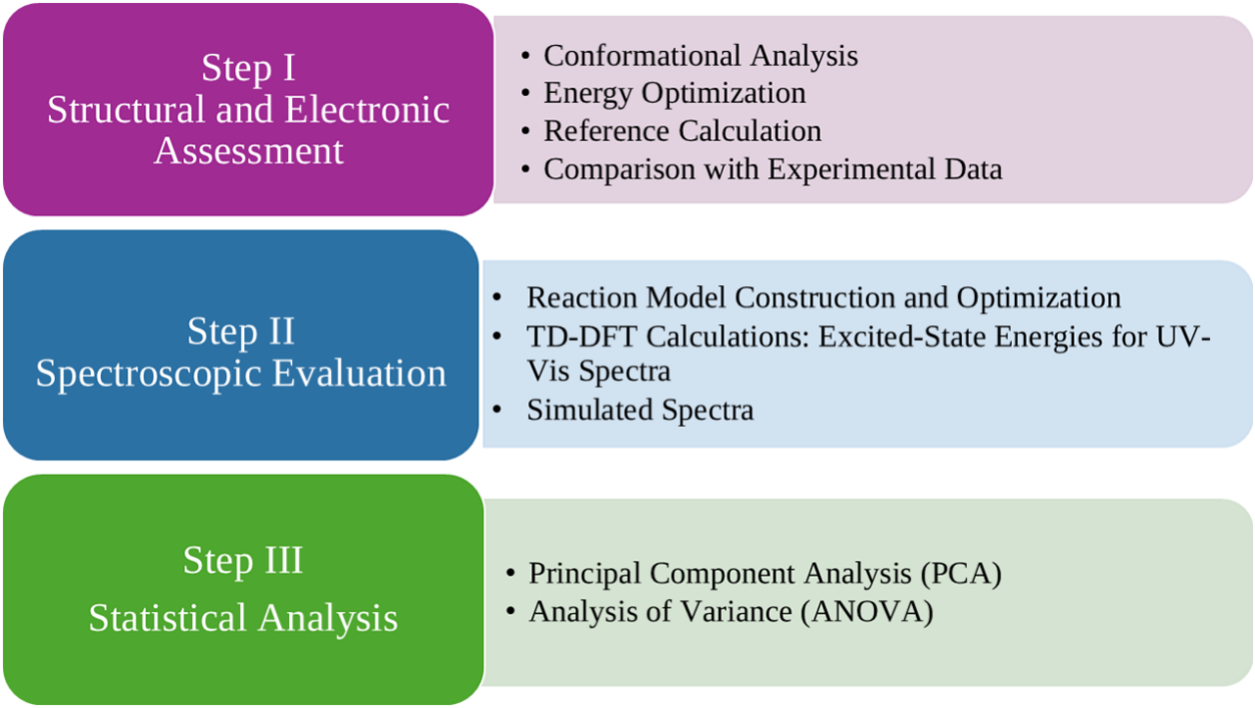
Schematic Diagram of the methodology.

## Results

### STEP I—Structural and Electronic Assessment

A systematic conformational analysis was conducted using the ORCA software, using the term Scan (Relaxed Surface Scan) to obtain the potential energy surface. To obtain greater surface detail, an angular increment of 5° was chosen, resulting in 72 structural conformers. All conformers were optimized at the B3LYP/TZVP level of theory.^58^ The dihedral chosen to be rotated was constituted by carbon atoms 1, 2, 3, and 4 (Figure 4A).

**Figure 4:**
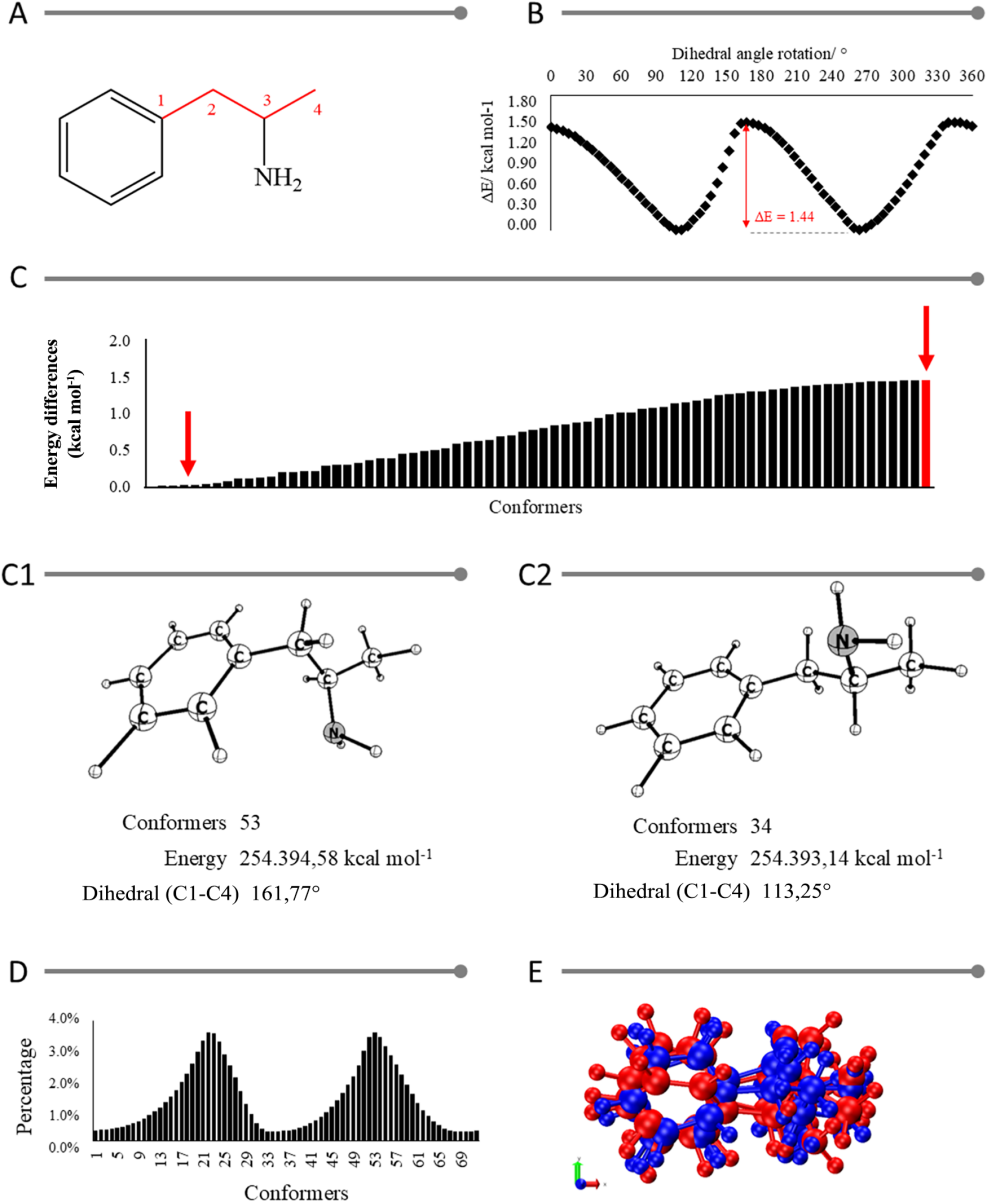
Structural and energetic evaluation of amphetamine (a01), with comparison with crystallographic structures deposited in databases, as follows: (A) Base structure for conformational analysis with the dihedral (C1–C2–C3-C4) indicated in red; (B) The energy curve of conformers originates from rotating the C1–C4 dihedral. The abscissa axis represents the difference in energy between the conformers with the lowest energy and each of the others; (C) Energy difference between the conformers obtained for the conformational analysis of amphetamine; (C1) the lowest and (C2) highest energy structures; (D) Graphical representation of the percentage relative to the Boltzmann distribution for the possible structural conformations of amphetamine; e (E) Overlay of the structures described in Table S2, where the molecules obtained experimentally (CCDC) are highlighted in blue and those obtained by computer simulation are highlighted in red.

The results obtained by conformational analysis allowed us to observe two regions of lower energy (Figure 4B). These regions indicate the structure’s isomers. The first region occurred between the 106° rotation and the second between 264°. The smooth curves are due to low steric hindrance and thus high rotation possibility. The difference between the lowest energy structure (conformer 53 with 264° rotation) and the highest (conformer 34 with 167° rotation) was 1.44 kcal mol^–1^. Figure 4C groups the energy differences between the lowest energy structure and all the other 72 conformers.

According to Figure 4C1,C2, the most abundant conformers were #23 (3.60%) and #53 (3.64%). The sum of the percentages of the other structures was 92.76%. The Boltzmann distribution analysis was performed using energy as a parameter to demonstrate that there is not enough energy difference between the conformers. This evaluation allowed us to determine the probability of observation of each structure, obtaining the graph represented in Figure 4D.

Based on these results, the evaluation continued to the structural analysis of bond lengths, angles, and dihedrals (Available in Supporting Information on Table S2). For this, the following were used: (i) structure #53 using the B3LYP/TZVP combination; (ii) structure #53 using the MP2/TZVP combination; (iii) the structure optimized in a previous work^22^ with B3LYP/6–31G**; and (iv) experimental structures available in the literature.^59–62^ The Cambridge Crystallographic Data Centre (CCDC) obtained the experimental structures. These were norephedrine (identifier JASTUS, number 1183066),^59^ norephedrine (identifier NOREPH01, number 1222608),^60^ cathine (identifier GEVNEB, number 617758)^62^ and amphetamine (identifier AMPETS02, number 729746).^61^ The superposition of these structures was represented in Figure 4E.

The experimental and simulated bond lengths and angles were very close, with no visible difference (Figure 4E). However, the dihedrals diverged significantly, indicating that the simulated and experimental structures may be isomers. Pearson’s correlation between the structures was applied to obtain more information from these data. Heat maps expressed the response (Figure 5A,B).^43^ The experimental data were identified as Exp. One (norephedrine–JASTUS), Exp. Two (norephedrine–NOREPH01), Exp. Three (cathine) and Exp. Four (amphetamine).

**Figure 5:**
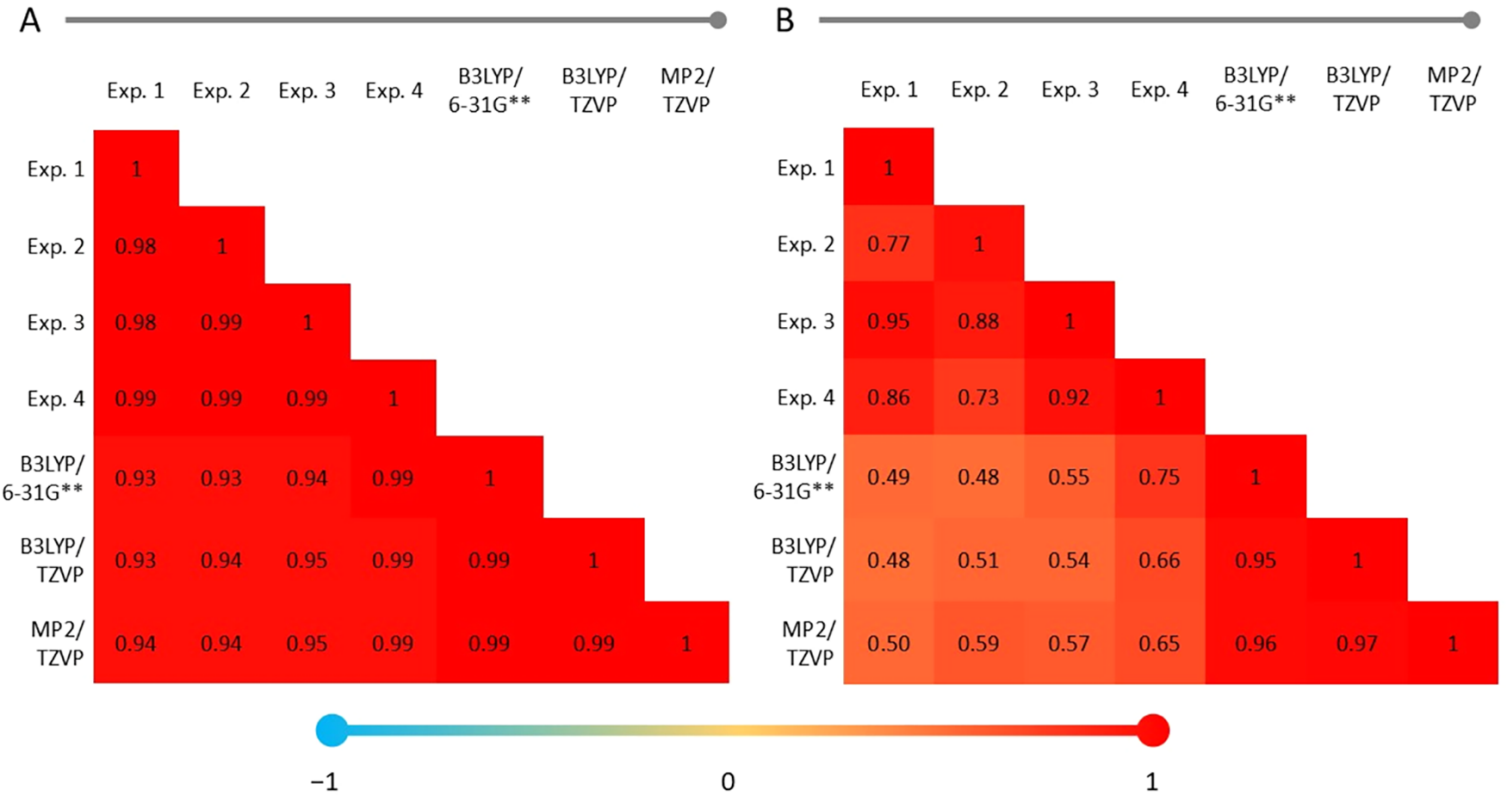
Heat map with Pearson correlation coefficients for (A) bond lengths and (B) bond angles.

Figure 5A shows the correlations regarding the bond lengths of each experimental and theoretical structure. There is a high similarity between the measurements, greater than 0.9248 (Exp. One and B3LYP/6–31G**). Removing the identical combinations from the comparison, the computational method most resembles the experimental data was the correlation between Exp. Four with B3LYP/6–31G** (0.9918). Figure 5B groups the correlation responses obtained for the bond angles studied based on the same procedure. The data for bond length showed greater divergence.

By combining the data related to bond angles, Figure 5B shows a greater divergence between the experimental and theoretical data. The most significant difference was in the correlation between Exp. One with B3LYP/TZVP (0.4751) and the combination Exp. Four with B3LYP/6–31G** (0.7497), which presented the most significant similarity between theoretical and experimental data. It can be noted that this last correlation (experimental versus computational) was greater than the response between Exp. Two and Exp. Four (experimental versus experimental); in addition, it was close to that observed for Exp. One with Exp. Two (experimental versus experimental).

Since the numerical values for dihedral angles already presented significant divergences (Table S2), it was understood that these are isomers. Thus, Pearson’s correlation was not applied, since the values would be wildly divergent and no helpful information could be extracted. Due to the computational cost and the correlation responses obtained in Figure 5, in addition to the conformational analysis indicating that there is no majority structure for amphetamines, the optimization of the other structures with and without FITC was performed with B3LYP/6–31G** without the presence of solvents.

To obtain the spectra in the ultraviolet–visible (UV–vis) region, TD-DFT B3LYP/6–31G** was used with the insertion of methanol (dielectric constant 32.63 and refractive index 1.329) and hexane (dielectric constant 1.89 and refractive index 1.375) as implicit solvents, with the continuous solvent method CPCM (conductor-like polarizable continuum model). Furthermore, we used the results obtained for the gas phase for this comparison. These solvents were chosen for their physical-chemical properties and because they are already indicated for this purpose. Table 1 shows the excitation energies for the molecules of the study system, isolated and bound to FITC, for each solvent.

**Table 1:** Absorption Energies (*E*_h_) for the Amphetamines Studied and Those Bound to FITC

	**gas phase**		**methanol**		**hexane**	
	ANF^a^	ANF + FITC^b^	ANF^a^	ANF + FITC^b^	ANF^a^	ANF + FITC^b^
a01	5.348	2.595	5.397	2.763	5.372	3.022
a02	5.161	2.606	5.247	2.775	5.201	3.033
a04	4.928	2.656	5.057	2.820	4.982	3.046
a05	5.037	2.702	5.089	2.866	5.058	3.041
a06	4.969	2.664	5.091	2.825	5.022	2.995
a07	4.906	2.653	5.043	2.818	4.966	3.002
a08	4.911	2.528	4.962	2.682	4.930	2.898
a09	4.868	2.670	4.892	2.834	4.871	2.942
a10	4.899	2.674	4.913	2.840	4.897	2.994
a11	4.724	2.688	4.846	2.854	4.778	3.076
a12	4.912	2.665	5.022	2.830	4.959	2.994
a13	4.991	2.662	5.086	2.818	5.032	2.987
a14	4.897	2.678	4.902	2.842	4.891	2.983

a Amphetamine.

b FITC-bound amphetamine.

The information in Table S2 allows us to analyze the fact that the bond with FITC decreases the excitation energy. Excitation energy is important information to be analyzed in the transitions that occur between the HOMO (highest busy molecular orbital) and LUMO (lowest unoccupied molecular orbital) orbitals. The first concerns electron donors and the second concerns acceptors.^63^ The smaller the energy difference between these orbitals (HOMO–LUMO or GAP), the more the absorption/emission will be shifted toward the visible spectrum. As the energy needed to excite electrons decreases, transitions move toward the visible region. However, some molecular systems may not have an adequate GAP for certain photophysical processes.^64^ Efficient reactions require adjacent orbitals or ones with enough energy.^65^ Therefore, more than one orbital may contribute to the excited state. The HOMO–LUMO difference in the gas phase for the studied system is shown in Table S3. When the substances in the Study System react with FITC, the resulting compound needs less energy to excite an electron to the next unoccupied orbital, leading to observable visual phenomena (if the transition is π→π* or *n*→π*).^66^ Therefore, it is understood that there is an approximation of the energy of the orbitals as shown in Figure 6, considering all solvents used in this study. Amphetamines are depicted in shades of blue across the different solvents, while those bound to FITC are shown in shades of red.

**Figure 6:**
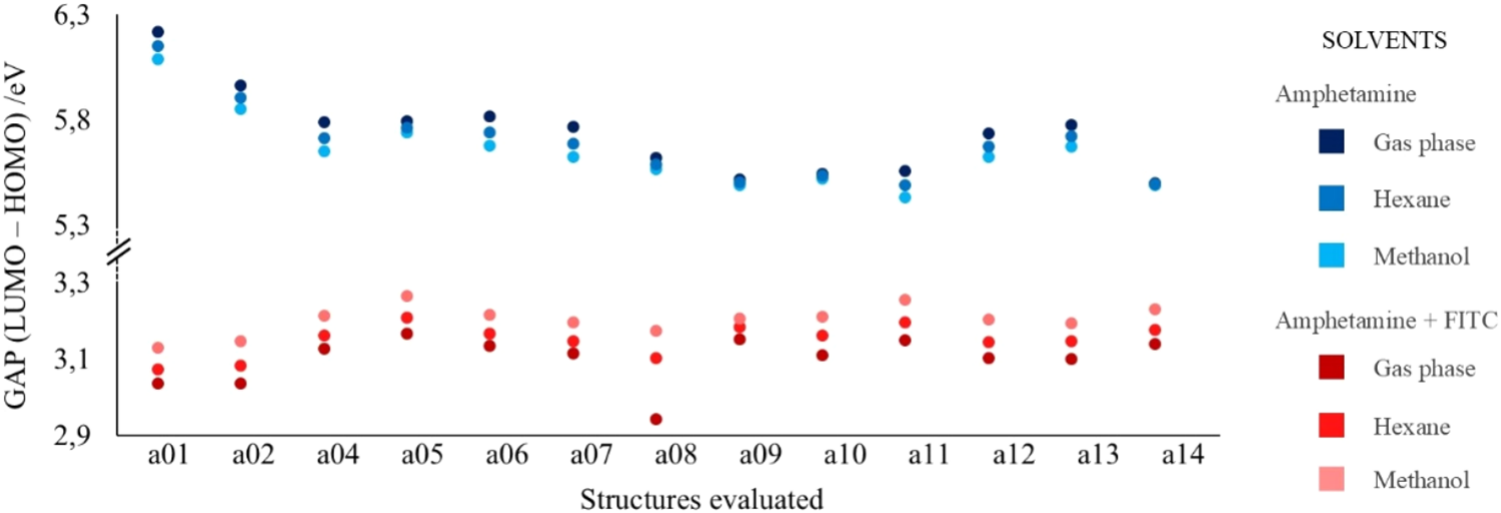
Scheme representing the energy differences between the HOMO and LUMO orbitals for the different solvents between amphetamines (tons of blue) and those bound to FITC (tons of red).

Analyzing Figure 6 and the data in Table S3 (Supporting Information), it can be seen that the average value for the HOMO–LUMO transitions of the amphetamines studied was 5.918 eV (136.47 kcal mol^–1^) and for those bound to FITC, it was 3.2040 eV (73.89 kcal mol^–1^). This energy decrease results in the absorption band’s displacement to a less energetic region. Consequently, this band becomes closer to the visible region, and thus, a blue coloration can be observed in experimental tests.^26,28,67^

### STEP II—Spectroscopic Evaluation

A standard or Gaussian distribution of the values was used for the spectroscopic evaluation, as the output files offer well-defined peaks and the data do not have the same dimension. Table 2 contains the maximum wavelengths for the molecules in the study system and data relating to the oscillator strength (*f* ^osc^). This can be understood as an analogue to the molar absorptivity (ε).^68,69^

**Table 2:** Maximum Wavelength (λ_máx_) and Oscillator Strength (*f* ^osc^) for Each Amphetamine Present in the Study System in Each Chemical Environment Studied

	**gas phase**		**methanol**		**hexane**	
molecules	λ_máx_/nm	*f*^osc^/au	λ_máx_/nm	*f*^osc^/au	λ_máx_/nm	*f*^osc^/au
a01	162.30	0.784	166.70	1.148	167.80	0.925
a02	160.40	0.545	167.20	1.049	165.60	0.712
a04	162.30	0.795	166.70	1.226	167.50	1.237
a05	162.70	0.669	168.20	0.655	168.60	1.111
a06	165.30	1.279	169.90	1.325	170.70	1.414
a07	166.50	1.182	170.90	1.489	171.70	1.524
a08	168.40	0.858	172.40	1.053	173.30	1.052
a09	174.50	0.529	179.60	0.795	178.30	0.503
a10	174.60	0.473	179.30	0.889	180.20	0.768
a11	161.10	0.419	166.90	1.271	167.70	1.255
a12	161.90	0.401	166.80	1.221	167.60	1.258
a13	165.40	1.084	170.00	1.484	170.80	1.496
a14	173.30	0.489	177.60	0.560	178.80	0.845
a15	175.70	0.587	179.20	0.894	180.00	0.774
a16	165.20	0.165	168.10	0.903	168.60	1.090
a17	166.50	0.683	170.40	0.993	171.10	1.045
a18	174.80	0.451	179.50	0.817	180.10	0.796
a19	175.60	0.743	179.30	0.899	179.80	0.882
a20	178.20	0.192	179.70	0.822	180.40	0.683
a21	194.10	1.301	202.00	2.244	203.10	2.253

The maximum wavelengths observed in Table 2, the λ_máx_, ranged from 160.40 nm for a02 in the gas phase to 203.10 nm for a21 in hexane. Furthermore, a similar λ_máx_ behavior is observed among the structures that have substituent groups: (i) alkyl (a01, a02, a04, a05, a06, a07, a11, a12, a13, a16 and a17); (ii) benzodioxole (a09, a10, a14, a15, a18, a19 and a20); and (iii) β-naphthyl (a21). These observations can be applied to all cases of solvents evaluated. Figure 7A represents the spectrum obtained for amphetamine (a01), the spectrum of FITC, and a01 bound to FITC, all in the gas phase. Amphetamines have their absorption spectrum in the ultraviolet region (100–400 nm). After the reaction, the spectrum shifts to the beginning of the visible region (Figure 7A) due to the presence of the FITC chromophore group.

**Figure 7:**
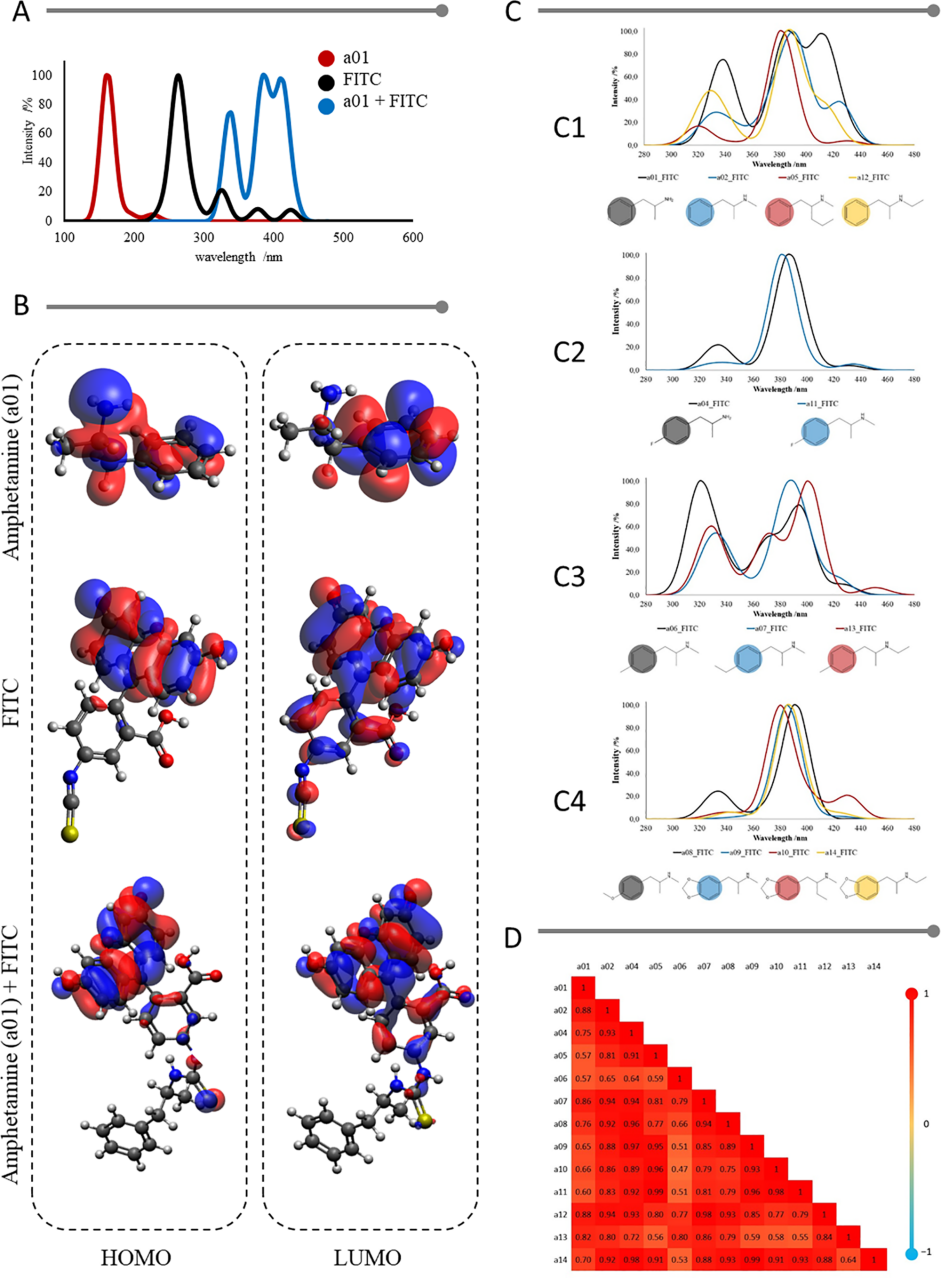
Spectroscopic evaluation of amphetamine (a01) and its homologues when they react with the FITC indicator, being: (A) Simulated absorption spectra obtained for amphetamine (in red), FITC alone (in black), and amphetamine bound to FITC (in blue); (B) Representation of the HOMO and LUMO molecular orbitals for amphetamine, FITC and the product of the reaction between them; (C) Combination of the spectra of amphetamines with FITC in the range of 280 to 480 nm for the gas phase, being (C1) only alkyl groups; (C2) alkyl and halogen groups; (C3) alkyl groups replacing the unsaturated chain as the aromatic ring; and (C4) alkyl groups and oxygenated groups (methoxyl and benzodioxole); and (D) Heat map with Pearson correlation coefficients for the spectra of FITC-bound amphetamines in the gas phase.

Figure 7B shows the orbitals (HOMO and LUMO) for amphetamine, FITC and the thiourea bond between them. The trend observed in the orbitals was that after binding with amphetamines, the HOMO orbitals of the reaction product resemble the LUMO orbital of pure FITC. This result shows that the small bands of fluorescein (Figure 7A) were activated and absorbed in the visible. Thus, absorption occurs closer to the near-ultraviolet and visible regions.

Figure 7C shows the spectra for all structures evaluated and a trend of two wavelength peaks, one between 280 and 350 nm and the other between 350 and 480 nm. As observed by the orbitals and highlighted in Table 2, the first band may indicate the probable absorption of sulfur from FITC and/or alkyl groups present in the structures in the *n* → σ*. transition. The second is understood as an activation of FITC, which provided π → π* and/or *n* → π* transitions.

Pearson’s correlation was used to obtain its coefficient and evaluate the similarity between the spectra illustrated in Figure 7C. With this data, a heat map (Figure 7D) was constructed to visually analyze the divergences between the spectra. Figure 7D summarizes the responses to the correlation analyses of the complete spectra, not just for λ_máx_, as in Table 2. The correlations ranged from 0.29 for a01–a15 to 0.99 for a05–a11 and a09–a14. Furthermore, this figure provides a more comprehensive evaluation of the spectrum and additional information on the structure-spectrum relationship.

In addition to verifying the correlation between the spectra using Pearson’s correlation coefficient (Figure 7D), we also assessed whether there were statistical differences within each group. For this verification, we used the ANOVA (analysis of variance) statistical method to determine whether there were significant differences between the means of independent groups. The null hypothesis (H_0_) indicates that there is no relationship between the spectra. On the other hand, the alternative hypothesis (H_1_ or Hα) suggests that the observations are influenced by some nonrandom factor (rejecting the null hypothesis). A significance level of 0.05 was used for this test. Table 3 shows the ANOVA results, where *df* is the degrees of freedom, *F* is the value obtained in the *F* test with its respective critical value (*F* critic), and the *P*-value.

**Table 3:** Evaluation of the Spectral Groups of Amphetamines and Cathinones Using ANOVA Statistics (Single Factor) in the Different Simulated Solvents

	**solvent**	**d** ***f*** **(between + within groups)**	***F***	***P*-value**	***F* critic**
amphetamine-likes	gas phase	713 (13 + 700)	2.26	0.0066	2.21
	methanol		0.37	0.9775	
	hexane		1.31	0.2028	
cathinone-likes	gas phase		0.50	0.9249	
	methanol		0.21	0.9985	
	hexane		1.37	0.1666	

Interpreting the ANOVA (single factor) results presented in Table 3, we observe that the *P*-value was greater than the significance level (*P* = 0.05); thus, we understand that there is no evidence to reject the null hypothesis. Therefore, the spectra for amphetamines in methanol and hexane are statistically identical, with 95% confidence. The same observation can be made for cathinones in all solvents studied. The *F*-test also supports this observation, as the calculated *F* (*F*) was smaller than the critical *F* (*F* critic). On the other hand, the amphetamine molecules without the insertion of an implicit solvent (gas phase) indicated that not all spectra are identical within the group. However, extrapolating to a real-world scenario, some solvent would be present, so it is understood that, overall, there is no evidence to indicate that the results are different.

### STEP III—Statistical Evaluation of Amphetamines and Cathinones

Similarly to that presented in Figure 7D, the Pearson correlation coefficient was calculated, with the subsequent heat map, for the spectra of amphetamines and cathinones (Figure 8).

**Figure 8:**
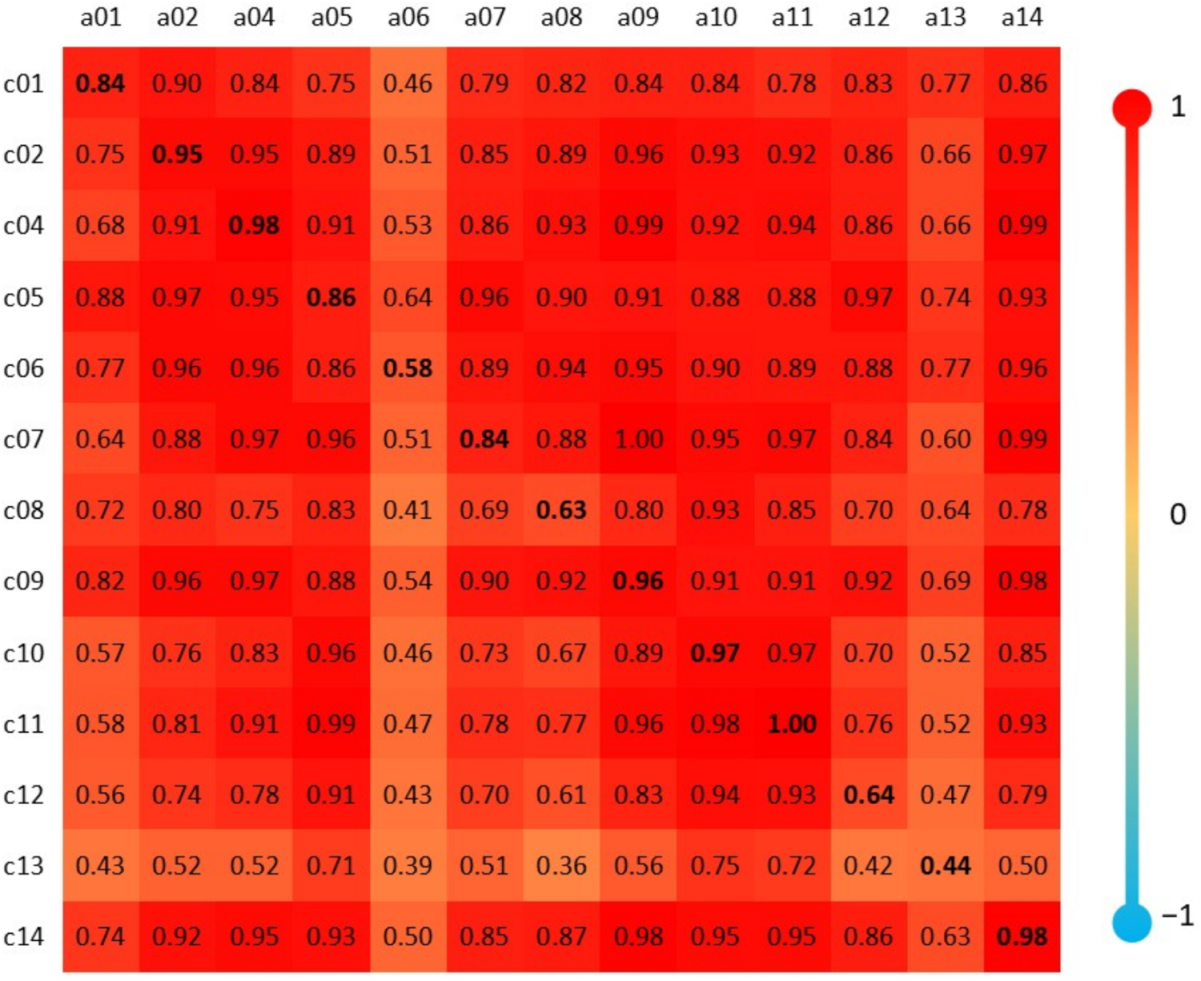
Heat map with Pearson correlation coefficients between the spectra of amphetamines and homologous cathinones bound to FITC for the gas phase.

In Figure 8, the diagonal line indicating the homologous structures between amphetamines and cathinones stands out. The lowest correlation between them was 0.44 between a13–c13, and the highest was 1.00 between a11–c11. Among the nonhomologous structures, the lowest correlation occurred between a08–c13 (0.36) and the highest was between a09–c07 (1.00). However, there is no well-defined pattern that objectively indicates amphetamines and cathinones.

A multivariate evaluation is necessary to evaluate this effect and observe the natural relationship between the amphetamine and cathinone data. An exploratory analysis, Principal Component Analysis, was performed on the 51 variables (wavelengths) and 28 samples (14 amphetamines and 14 cathinones bound to FITC). Due to the nature of the data, no pretreatment was applied.

Figure 9 groups the scores, representing the samples rewritten in the new principal component system (CP or Factor). The structures of amphetamines with FITC are indicated in black, and the cathinones with FITC are noted in red.

**Figure 9:**
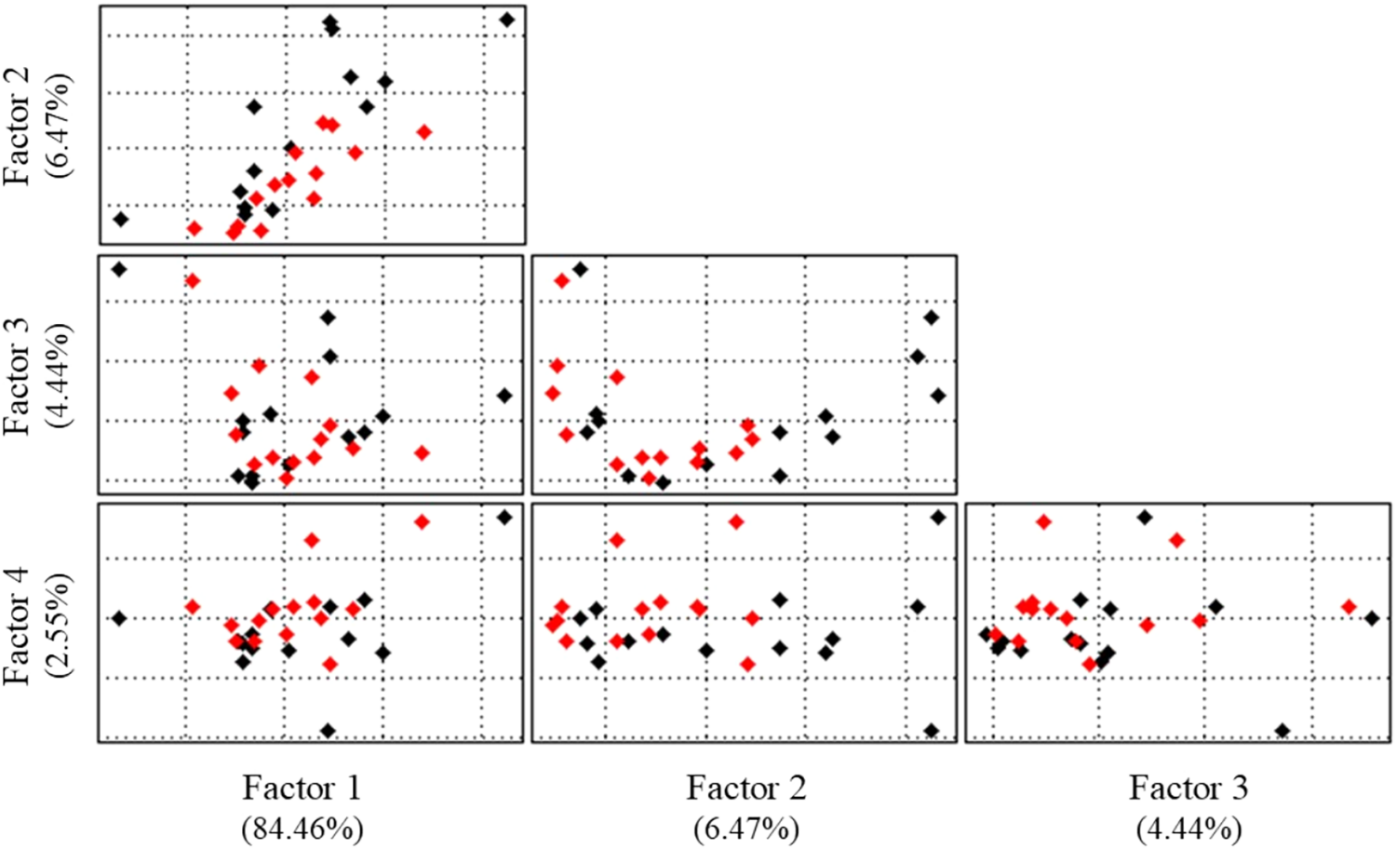
PCA results in unsupervised evaluation of the spectra in the UV–vis region.

Each of the 51 variables can be interpreted as a dimension. Thus, after PCA, all the information in the original data system was reduced to fewer principal components (Figure 9). Cumulatively, Factor 1 contains 84.4576% of all the original information, Factor 2 results in 90.9276%, Factor 3 reaches 95.3628%, and the sum of the other Factors represents 4.6372%.

Additionally, an ANOVA test was performed to assess whether there would be a significant difference between all the spectra. This analysis was performed to ensure that there was indeed no possibility of differentiating the entire set of spectra with a confidence level of 0.05. To this end, we used the same approach as already presented for each of the classes (Table 3). When performing the test, 1427 degrees of freedom were obtained (27 between and 1400 within groups), and the respective *F*-critical value was 1.67. We obtained values of 1.42 (gas phase), 0.29 (methanol), and 1.32 (hexane) for the calculated *F*, and 0.0768 (gas phase), 0.9999 (methanol), and 0.1293 (hexane) for the *P*-value. Interpreting these results, again, there is no evidence to reject the null hypothesis (The null hypothesis indicates that there is no relationship between the spectra).

## Discussions

Some colorimetric tests are more specific than others, and some pose a risk to the professional who performs them. Philp & Fu^9^ demonstrated the main tests for illegal drugs performed, as well as their composition and dangerousness. In the current scenario, where a series of spot tests are being used for a wide range of drugs of abuse, the search for specific and selective reactions for the preliminary detection of these substances is a desired target.

In this study, the structural and spectroscopic properties were evaluated through theoretical chemistry. The conformers and the responses obtained from the Boltzmann distribution indicated that the most likely structure by conformational analysis (Figure 4C1) does not come close to the crystallographic structures (Exp. One to Exp. Four). This occurred because the chiral carbon has sufficient free rotation as observed in the conformational analysis. In addition, we know that crystallographic structures can have intermolecular interactions that favor a geometry different from that observed in solution or the gas phase. For these reasons and because the energy difference between all conformers is insignificant, we used the structure with the lowest energy and not the crystal available in the literature. The computational approach that most closely matched the experimental responses was the B3LYP/6–31G** combination for Exp. Four.

Furthermore, we demonstrated that the reaction between drugs evaluated in this study (amphetamines and cathinones) and FITC is viable.^67^ The product of this reaction has a lower energy, between the HOMO and LUMO orbitals, than amphetamine before the response due to the bond with the chromophore group (FITC). As shown in Table 2 represent the tendency to be mainly the π → π* transition due to the aromatic ring with its double bonds (C=C).^70^ The transitions between the HOMO–LUMO orbitals present higher values for free amphetamines than for those bound to FITC, which was reflected in the λ_máx_ values. It is worth highlighting that the presence of halogen did not impact the λ_*máx*_, as in the case of amphetamines a04 and a11, and the presence of the methoxyl group (a08) provided a borderline behavior between compounds with only alkyl substituents and others with benzodioxole (Figure 7C).

Furthermore, the results for λ_máx_ of methanol were lower than for hexane. This effect is due to some medium characteristics, such as geometric characteristics and polarity of the solute. These parameters affect the spectra, since λ_máx_ is expected to occur at shorter wavelengths when the solvent is polar (water, alcohols, among others) and the molecule of interest is polar.^71^ Given this, we understand that alkyl and benzodioxole groups tend to be similar in analysis (Figure 7C). However, amine substituents are also important for correlations: alkyl chains introduced spectral divergences previously unseen. Additionally, the chemical environments provided more specific correlations, influenced by dielectric constants and polarity.

When analyzing the spectral data using Pearson’s correlation (univariate method—Figure 8) and the score results (Figure 9), we understand that no relationship can differentiate amphetamines from cathinones. The correlation coefficients were diffuse, and no objective standard for interpretation was established. A similar observation occurred for PCA, and there may be confusion between the results.

Since it is a colorimetric test, FITC could be used as a verification test when seizing substances suspected of containing amphetamines or cathinones. However, there was no clear definition between these classes of substances. There may be different reasons for this observation, such as (i) the similarity in the λ_*máx*_, regions, as indicated in Table 2; (ii) the presence of similar chromophore groups between the homologous structures; or (iii) the inability of FITC to perform this differentiation. Therefore, the analysis of the emission results becomes uncertain.

The use of FITC for identifying amphetamines and cathinones has been investigated in two studies.^53,67^ Lloyd et al.^67^ created a microchip capable of detecting these substances using FITC as a fluorophore. This study involved structures similar to ours, including five cathinones (4 mmc–c06, bkmdma–c09, bkmbdb–c10, nec–c12, 4mec–c13) and one amphetamine (nea–a12). They demonstrate that FITC and luminescent decay, combined with microfluidic technology (Agilent 2100 Bioanalyzer), can identify actual drug pill samples. Skultety et al.^53^ examined 43 cathinone derivatives and identified the electromagnetic spectrum range from 125 to 225 nm as key for differentiating the substances. The experimental spectral behavior and the computational data show consistent trends.

In practical terms, we were demonstrated that FITC may not be the most assertive choice for a preliminary test, but it can be used in a cascade of colorimetric screening assays.^18,72^ When conducting this study, the limitation of FITC was identified, as it reacts only with primary and secondary amines, not considering tertiary amines. As an observation for other studies focused on colorimetric tests, the study does not need to be limited to studying only the substances, but also to consider possible adulterants and/or contaminants that may also react.^73^ Based on these values (*F*-test and *P-*value) shown in Table 3 and previously (STEP III—Statistical evaluation of amphetamines and cathinones), it is understood that the absence of a clustering profile in the PCA (Figure 9) and the correlations observed in Figure 8 are associated with the similarity in the responses. Thus, chemically and statistically, it is not possible to observe a clear definition between amphetamines and cathinones using FITC as a preliminary test.

Finally, as the number of NPS reports grows each year, alternative colorimetric tests might need new studies or evaluations. In silico methods could offer a quicker, more cost-effective option. This study showed this by providing insights into whether reactions are viable. Additionally, it enabled the analysis of other NPS structures that had not yet been examined reported.

## Conclusions

The computational methods proved capable of identifying the lowest-energy structure for amphetamine. Due to the low steric hindrance, the structure has rotational flexibility. Furthermore, the responses to the computational tests were consistent with the experimental results. In addition to the three-dimensional structure of amphetamine, the possibility of using the modified fluorescein indicator, FITC (fluorescein isothiocyanate), for preliminary detection of this class of drugs was studied. The results obtained in this part of the work demonstrate that it is possible to make a theoretical assessment that the reaction of FITC with the groups of molecules studied is feasible.

When the data were used to identify amphetamines, it was observed that there was little distinction between the different structures based on their spectra, even when a mixture of them was present, which could be noted by overlapping spectra.

However, it is important to highlight that B3LYP may not be the best choice for describing the excited states because this functional lacks long-range correction, making charge transfer transitions poorly defined. What can happen when comparing vertical excitations – between experimental and computational data – is a similarity due to error cancellation. We emphasize that if the excited state is optimized to obtain adiabatic transitions, the correlation may worsen. Therefore, it is recommended that other studies use a functional with long-range correction, such as CAM-B3LYP and wB97XD. Additionally, it is advisible to explicitly consider the solvent effect. Given the purpose of this work — to demonstrate trends in conformational and energetic analysis, spectroscopic characterization, and statistical comparison — it is concluded that in silico methods can provide information for substances that are still unknown or poorly documented. Using these techniques can help optimize the collection of information and serve as a useful tool in forensic investigations.

## Supplementary Material



## Notes

File with chemical structures, molecular formulas, CAS number and nomenclatures (IUPAC, common and synonyms) for the 42 molecules evaluated in the study (PDF)

## References

[1] United Nations Office on Drugs and Crime - UNODC . Current NPS Threats, 1st ed.; United Nations publication: Vienna, 2020; Vol. III pp 1–6. https://www.unodc.org/documents/scientific/Current_NPS_Threats_Vol.3.pdf (accessed Aug 2, 2025).

[2] Korf D., Benschop A., Werse B., Kamphausen G., Felvinczi K., Dabrowska K., Hernriques S., Nabben T., Wieczorek L., Bujalski M., Kalo Z., Hearne E., van Hout M. C. (2019). How and
Where to Find NPS Users:
A Comparison of Methods in a Cross-National Survey Among Three Groups
of Current Users of New Psychoactive Substances in Europe. Int. J. Mental Health Addict..

[3] United Nations Office on Drugs and Crime - UNODC . Current NPS Threats; Vienna, 2024, pp. 1–6. https://www.unodc.org/documents/scientific/Current_NPS_threats_VII.pdf (accessed Aug 2, 2025).

[4] Brensilver M., Heinzerling K. G., Shoptaw S. (2013). Pharmacotherapy of Amphetamine-Type
Stimulant Dependence: An Update. Drug Alcohol
Rev..

[5] United Nations Office on Drugs and Crime . Drug Market Trends: Cocaine, Amphetamine-Type Stimulants, New Psychoactive Substances. In World Drug Report 2022; United Nations Office on Drugs and Crime , Ed.; United Nations publication: Vienna, 2022; Vol. Booklet 4, pp 1–111.

[6] Dragan A.-M., Parrilla M., Feier B., Oprean R., Cristea C., de Wael K. (2021). Analytical Techniques for the Detection of Amphetamine-Type
Substances in Different Matrices: A Comprehensive Review. TrAC, Trends Anal. Chem..

[7] Heal D. J., Smith S. L., Gosden J., Nutt D. J. (2013). Amphetamine, Past
and Present – a Pharmacological and Clinical Perspective. J. Psychopharmacol..

[8] Scientific Working Group for the Analysis of Seized Drugs, (SWGDRUG) . SWGDRUG Recommendations Version 7.1; United States Departament of Justice: Washington DC, USA, 2016.

[9] Philp M., Fu S. (2018). A Review of Chemical
‘Spot’ Tests: A Presumptive Illicit
Drug Identification Technique. Drug Test. Anal..

[10] Nagy, G. ; Szöllösi, I. ; Szendrei, K. Colour Tests for Precursor Chemicals of Amphetamine-Type Substances UNODC: SCIENTIFIC AND TECHNICAL NOTES 2005, No. December, pp 1–17.

[11] Pandey S., Borders T. L., Hernández C. E., Roy L. E., Reddy G. D., Martinez G. L., Jackson A., Brown G., Acree W. E. (1999). Comparison
of Analytical Methods: Direct Emission versus First-Derivative Fluorometric
Methods for Quinine Determination in Tonic Waters. J. Chem. Educ..

[12] O’Reilly J. E. (1975). Fluorescence
Experiments with Quinine. J. Chem. Educ..

[13] Kolmakov K., Belov V. N., Bierwagen J., Ringemann C., MÃ1/4ller V., Eggeling C., Hell S. W. (2010). Red-Emitting
Rhodamine
Dyes for Fluorescence Microscopy and Nanoscopy. Chem. - Eur. J..

[14] Boens N., Leen V., Dehaen W., Wang L., Robeyns K., Qin W., Tang X., Beljonne D., Tonnelé C., Paredes J. M., Ruedas-Rama M. J., Orte A., Crovetto L., Talavera E. M., Alvarez-Pez J. M. (2012). Visible Absorption and Fluorescence
Spectroscopy of Conformationally Constrained, Annulated BODIPY Dyes. J. Phys. Chem. A.

[15] Shishkanova T. V., Vatrsková L., Spálovská D., Králík F., Cuřínová P., Winkler M., Budka J., Jurásek B., Kuchař M., Setnička V. (2020). Complexation
of Cathinones by 4-Tert-Butylcalix[4]­Arene Tetra-Acetate as a Possible
Technique for Forensic Analysis. Forensic Toxicol..

[16] Kellett K., Broome J. H., Zloh M., Kirton S. B., Fergus S., Gerhard U., Stair J. L., Wallace K. J. (2016). Small Molecule Recognition
of Mephedrone Using an Anthracene Molecular Clip. Chem. Commun..

[17] Hermanson, G. T. Fluorescent Probes. In Bioconjugate Techniques; Elsevier, 2013; pp 395–463 10.1016/B978-0-12-382239-0.00010-8.

[18] Bruni A., Rodrigues C., dos Santos C., de Castro J., Mariotto L., Sinhorini L. (2021). Analytical
Challenges for Identification
of New Psychoactive Substances: A Literature-Based Study for Seized
Drugs. Braz. J. Anal. Chem..

[19] UNODC . Recommended Methods for the Identification and Analysis of Synthetic Cathinones in Seized Materials; United Nations publication: New York, 2015.

[20] Brush, C. K. Fluorescein Labelled Phosphoramidites. 5,583,236, October 10, 1996. https://patentimages.storage.googleapis.com/6c/c7/d0/06b7d9212ce24c/US5583236.pdf (accessed Aug 2, 2025).

[21] Bruni A. T., de Carvalho P. O. M., Rodrigues C. H. P., Leite V. B. P. (2018). In Silico Methods
in Forensic Science: Quantum Chemistry and Multivariate Analysis Applied
to Infrared Spectra of New Amphetamine- and Cathinone-Derived Psychoactive
Substances. Forensic Chemistry.

[22] Rodrigues, C. H. P. ; Bruni, A. T. Estudos in Silico Do Comportamento de Catinonas Sintéticas Com Interesse Forense. In Dissertação (Mestrado); Universidade de São Paulo: Ribeirão Preto, 2019 10.11606/D.59.2019.tde-23102018-112244.

[23] Hermanson G. T. (2013). Chapter
10 – Fluorescent Probes. Bioconjugate
Tech..

[24] Marques, M. A. L. ; Ullrich, C. A. ; Nogueira, F. ; Rubio, A. ; Burke, K. ; Gross, E. K. U. Time-Dependent Density Functional Theory; Springer-Verlag, 2006.

[25] Runge E., Gross E. K. U. (1984). Density-Functional
Theory for Time-Dependent Systems. Phys. Rev.
Lett..

[26] Adamo C., Jacquemin D. (2013). The Calculations of Excited-State
Properties with Time-Dependent
Density Functional Theory. Chem. Soc. Rev..

[27] Chu G., Yang Z., Xi T., Xin J., Zhao Y., He W., Shui M., Gu Y., Xiong Y., Xu T. (2018). Relaxed Structure
of Typical Nitro Explosives in the Excited State: Observation, Implication
and Application. Chem. Phys. Lett..

[28] Cooper J. K., Grant C. D., Zhang J. Z. (2013). Experimental
and TD-DFT Study of
Optical Absorption of Six Explosive Molecules: RDX, HMX, PETN, TNT,
TATP, and HMTD. J. Phys. Chem. A.

[29] Tcharkhetian A. E. G., Bruni A. T., Rodrigues C. H. P. (2021). Combining Experimental and Theoretical
Approaches to Study the Structural and Spectroscopic Properties of
Flakka (α-Pyrrolidinopentiophenone). Results
Chem..

[30] Derks E. P. P. A., Beckers M. L. M., Melssen W. J., Buydens L. M. C. (1996). Parallel
Processing of Chemical Information in a Local Area Network—II.
A Parallel Cross-Validation Procedure for Artificial Neural Networks. Comput. Chem..

[31] Becke A. D. (1993). Density-functional
Thermochemistry. III. The Role of Exact Exchange. J. Chem. Phys..

[32] Peintinger M. F., Oliveira D. V., Bredow T. (2013). Consistent
Gaussian Basis Sets of
Triple-Zeta Valence with Polarization Quality for Solid-State Calculations. J. Comput. Chem..

[33] Tsuzuki S., Uchimaru T. (2020). Accuracy of Intermolecular
Interaction Energies, Particularly
Those of Hetero-Atom Containing Molecules Obtained by DFT Calculations
with Grimme’s D2, D3 and D3BJ Dispersion Corrections. Phys. Chem. Chem. Phys..

[34] Grimme S. (2012). Supramolecular
Binding Thermodynamics by Dispersion-Corrected Density Functional
Theory. Chem. - Eur. J..

[35] Grimme S., Ehrlich S., Goerigk L. (2011). Effect of the Damping
Function in
Dispersion Corrected Density Functional Theory. J. Comput. Chem..

[36] Grimme S., Antony J., Ehrlich S., Krieg H. (2010). A Consistent and Accurate
Ab Initio Parametrization of Density Functional Dispersion Correction
(DFT-D) for the 94 Elements H-Pu. J. Chem. Phys..

[37] Atkins, P. ; Jones, L. Cinética Química. In Princípios de Química: Questionando a vida moderna e o meio ambiente; Atkins, P. ; Paula, J. ; de Keeler, J. , Eds.; Alencastro, R. B. , Translator; Bookman: Porto Alegre, 2006; Vol. 1, pp 577–627.

[38] Lewin M. (2004). Solutions
of the Multiconfiguration Equations in Quantum Chemistry. Arch. Ration. Mech. Anal..

[39] Head-Gordon M., Pople J. A., Frisch M. J. (1988). MP2 Energy
Evaluation by Direct Methods. Chem. Phys. Lett..

[40] Head-Gordon M., Head-Gordon T. (1994). Analytic MP2 Frequencies without Fifth-Order Storage.
Theory and Application to Bifurcated Hydrogen Bonds in the Water Hexamer. Chem. Phys. Lett..

[41] Frisch M. J., Head-Gordon M., Pople J. A. (1990). A Direct MP2 Gradient Method. Chem. Phys. Lett..

[42] Hanwell M. D., Curtis D. E., Lonie D. C., Vandermeersch T., Zurek E., Hutchison G. R. (2012). Avogadro: An Advanced Semantic Chemical
Editor, Visualization, and Analysis Platform. J. Cheminf..

[43] Saccenti E., Hendriks M. H. W. B., Smilde A. K. (2020). Corruption of the Pearson Correlation
Coefficient by Measurement Error and Its Estimation, Bias, and Correction
under Different Error Models. Sci. Rep..

[44] Silva C., Braz A., Pimentel M. F. (2019). Vibrational Spectroscopy and Chemometrics
in Forensic Chemistry: Critical Review, Current Trends and Challenges. J. Braz Chem. Soc..

[45] Neese F. (2012). The ORCA Program
System. WIREs Comput. Mol. Sci..

[46] Neese F. (2018). Software Update:
The ORCA Program System, Version 4.0. WIREs
Comput. Mol. Sci..

[47] Frisch M.
J., Pople Ja., Binkley J. S. (1984). Self-Consistent Molecular Orbital
Methods 25. Supplementary Functions for Gaussian Basis Sets. J. Chem. Phys..

[48] van
Caillie C., Amos R. D. (2000). Geometric Derivatives of Density
Functional Theory Excitation Energies Using Gradient-Corrected Functionals. Chem. Phys. Lett..

[49] Casida M. E., Jamorski C., Casida K. C., Salahub D. R. (1998). Molecular Excitation
Energies to High-Lying Bound States from Time-Dependent Density-Functional
Response Theory: Characterization and Correction of the Time-Dependent
Local Density Approximation Ionization Threshold. J. Chem. Phys..

[50] Bauernschmitt R., Ahlrichs R. (1996). Treatment of Electronic
Excitations within the Adiabatic
Approximation of Time Dependent Density Functional Theory. Chem. Phys. Lett..

[51] Neese F., Wennmohs F., Hansen A., Becker U. (2009). Efficient, Approximate
and Parallel Hartree–Fock and Hybrid DFT Calculations. A ‘Chain-of-Spheres’
Algorithm for the Hartree–Fock Exchange. Chem. Phys..

[52] NIST . Standard Reference Database 101 NIST Computational Chemistry Comparison and Benchmark Database, National Institute of Standards and Technology 10.18434/T47C7Z

[53] Skultety L., Frycak P., Qiu C., Smuts J., Shear-Laude L., Lemr K., Mao J. X., Kroll P., Schug K. A., Szewczak A., Vaught C., Lurie I., Havlicek V. (2017). Resolution
of Isomeric New Designer Stimulants Using Gas Chromatography –
Vacuum Ultraviolet Spectroscopy and Theoretical Computations. Anal. Chim. Acta.

[54] Tominaga Y. (1999). Comparative
Study of Class Data Analysis with PCA-LDA, SIMCA, PLS, ANNs, and k-NN. Chemom. Intell. Lab. Syst..

[55] Ferreira, M. M. C. QUIMIOMETRIA - Conceitos, Métodos e Aplicações, 1st ed.; Editora da Unicamp: Campinas, 2015.

[56] Bruni A. T., Leite V. B. P., Ferreira M. M. C. (2002). Conformational
Analysis: A New Approach
by Means of Chemometrics. J. Comput. Chem..

[57] FáVero, L. P. L. ; Belfiore, P. P. Manual de Análise de Dados - Estatística e Modelagem Multivariada Com Excel, SPSS e Stata, 1st ed.; FáVero, L. P. L. ; Belfiore, P. P. , Eds.; Elsevier: Rio de Janeiro, 2017; Vol. 1.

[58] Rodrigues C. H. P., Leite V. B. P., Bruni A. T. (2021). Can NMR Spectroscopy Discriminate
between NPS Amphetamines and Cathinones? An Evaluation by in Silico
Studies and Chemometrics. Chemom. Intell. Lab.
Syst..

[59] Egli M., Dobler M. (1989). Structural Aspects
of the Enantioselectivity of Tartrates
with ?-Amino-Alcohol Salts. Part II. Crystal Structures of
(1R, 2S)-Norephedrine Hydrochloride and (1R, 2R)- Norpseudoephedrine
Hydrochloride. Helv. Chim. Acta.

[60] Mukhopadhyay B. P., Dattagupta J. K., Evrard G. H. (1991). Crystal and Molecular Structure of
(?)-1-Phenyl-2-Amino-1-Propanol, C9H13NO. J.
Crystallogr. Spectrosc. Res..

[61] Pogorzelec-Glaser K., Kaszyńska J., Rachocki A., Tritt-Goc J., Piślewski N., Pietraszko A. (2009). The Crystal Structure and Evidence
of the Phase Transition in D-Amphetamine Sulfate, as Studied by X-Ray
Crystallography, DSC and NMR Spectroscopy. New
J. Chem..

[62] Yamaguchi K., Makino Y., Urano Y., Nagano T. (1999). Absolute Configuration
of Cathine. Anal. Sci..

[63] Salim A. S., Girgis A. S., Basta A. H., El-saied H., Mohamed M. A., Bedair A. H. (2018). Comparative DFT
Computational Studies with Experimental
Investigations for Novel Synthesized Fluorescent Pyrazoline Derivatives. J. Fluoresc..

[64] Fukui K. (1982). The Role of
Frontier Orbitals in Chemical Reactions. Angew.
Chem., Int. Ed..

[65] Pereira D. H., Porta F. A. La., Santiago R. T., Garcia D. R., Ramalho T. C. (2016). New Perspectives
on the Role of Frontier Molecular Orbitals in the Study of Chemical
Reactivity: A Review. Rev. Virtual Química.

[66] Lacerda
Júnior V., Oliveira K. T. de., Silva R. C. E., Constantino M. G., Silva G. V. J. da. (2007). Reatividade Em Reações de Diels-Alder:
Uma Prática Computacional. Quim. Nova.

[67] Lloyd A., Russell M., Blanes L., Somerville R., Doble P., Roux C. (2014). The Application of Portable Microchip
Electrophoresis for the Screening and Comparative Analysis of Synthetic
Cathinone Seizures. Forensic Sci. Int..

[68] Beard E. J., Sivaraman G., Vázquez-Mayagoitia Á., Vishwanath V., Cole J. M. (2019). Comparative Dataset of Experimental and Computational
Attributes of UV/Vis Absorption Spectra. Sci.
Data.

[69] Westermayr J., Marquetand P. (2021). Machine Learning for Electronically
Excited States
of Molecules. Chem. Rev..

[70] Atkins, P. ; Paula, J. ; Keeler, J. Electronic Spectra. In Physical Chemistry; Atkins, P. ; Paula, J. ; Keeler, J. , Eds.; Oxford University Press: Oxford, 2018; Vol. 1, pp 459–469.

[71] Goes-Filho, L. da S. CARACTERIZAÇÃO E ESTUDOS CINÉTICOS DE ALBUMINA TRATADA COM ESPÉCIES REATIVAS DERIVADAS DE ÓXIDOS DE NITROGÊNIO: ESPECTROSCOPIA DE ABSORÇÃO E FLUORESCÊNCIA. In Dissertação (Mestrado); Pontifícia Universidade Católica do Rio de Janeiro (PUC-RIO): Rio de Janeiro, Brasil, 2005 10.17771/PUCRio.acad.8015.

[72] Toole K. E., Fu S., Shimmon R. G., Kraymen N., Taflaga S. (2012). Color Tests for the
Preliminary Identification of Methcathinone and Analogues of Methcathinone. Microgram J..

[73] Cole C., Jones L., McVeigh J., Kicman A., Syed Q., Bellis M. (2011). Adulterants in Illicit Drugs: A Review of Empirical
Evidence. Drug Test. Anal..

