# Supplementary material for: Computational Evaluation of the Use of Fluorescein Isothiocyanate as a Preliminary Test for Amphetamines and Cathinones
Source: ACS Omega. 2025 Aug 12;10(33):37849–61. doi: 10.1021/acsomega.5c04896 (PMC12391928; doi:10.1021/acsomega.5c04896)
Supplement: Supplementary file 1 [file ao5c04896_si_001.pdf]

SUPPORTING INFORMATION

Computational evaluation of the use of fluorescein isothiocyanate as a preliminary test for amphetamines and cathinones

Caio Henrique Pinke Rodrigues, Aline Thais Bruni *

Departmento de Química, Faculdade de Filosofia, Ciências e Letras de Ribeirão Preto, Universidade de São Paulo. Avenida Bandeirantes, 3900 - CEP 14040-901. Ribeirão Preto - SP -Brazil

*aline.bruni@usp.br (corresponding author's email)

ORCID ID

Aline Thais Bruni: https://orcid.org/0000-0002-7721-3042

Caio Henrique Pinke Rodrigues: https://orcid.org/0000-0002-7794-7484

Table S1. Amphetamines and cathinones studied in this work.

Code	Structure	Formula	CAS	IUPAC nomenclature	Common name	Synonyms
**a01**	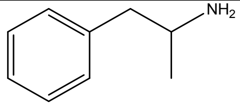	$C_9H_{13}N$	300-62-9	1-phenylpropan-2-amine	Amphetamine	-
**a02**	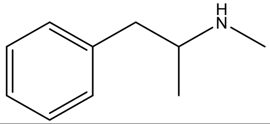	$C_{10}H_{15}N$	537-46-2	N-methyl-1-phenylpropan-2-amine	Methamphetamine	N-methylamphetamine, Crystal
**a03**	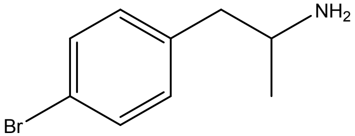	$C_9H_{12}BrN$	18455-37-3	1-(4-bromophenyl)propan-2-amine	p-Bromoamphetamine	PBA or 4-*bromoamphetamine*
**a04**	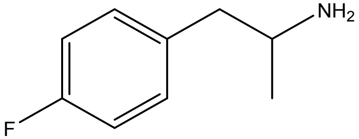	$C_9H_{12}FN$	459-02-9	1-(4-fluorophenyl)propan-2-amine	4-Fluoroamphetamine	4-FMP, PAL-303 or "*Flux*"
**a05**	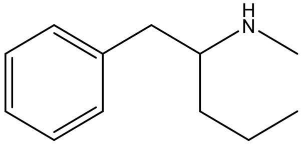	$C_{12}H_{19}N$		N-methyl-1-phenylpentan-2-amine	-	-
**a06**	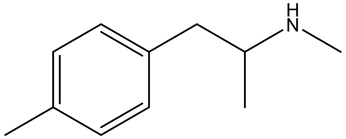	$C_{11}H_{17}N$		N-methyl-1-(p-tolyl)propan-2-amine	-	-
**a07**	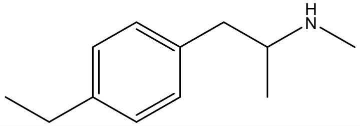	$C_{12}H_{19}N$	16048-29-6	1-(4-ethylphenyl)-N-methylpropan-2-amine	-	-

**a08**	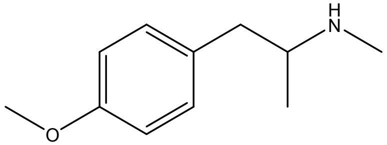	$C_{11}H_{17}NO$	22331-70-0	1-(4-methoxyphenyl)-N-methylpropan-2-amine	-	-
**a09**	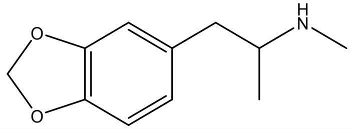	$C_{11}H_{15}NO_2$	42542-10-9	1-(benzo[d][1,3]dioxol-5-yl)-N-methylpropan-2-amine	-	-
**a10**	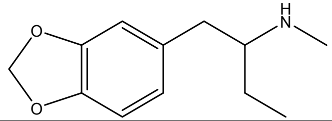	$C_{12}H_{17}NO_2$	103818-46-8	1-(benzo[d][1,3]dioxol-5-yl)-N-methylbutan-2-amine	-	-
**a11**	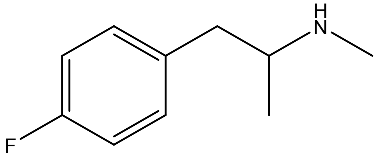	$C_{10}H_{14}FN$	351-03-1	1-(4-fluorophenyl)-N-methylpropan-2-amine	-	-
**a12**	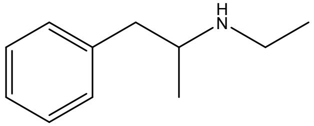	$C_{11}H_{17}N$	457-87-4	N-ethyl-1-phenylpropan-2-amine	-	-
**a13**	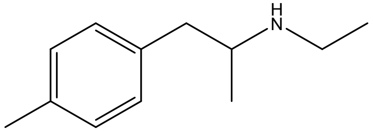	$C_{12}H_{19}N$		N-ethyl-1-(p-tolyl)propan-2-amine	-	-
**a14**	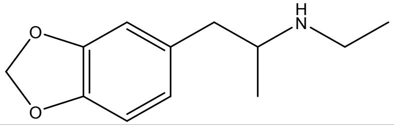	$C_{12}H_{17}NO_2$	82801-81-8	1-(benzo[d][1,3]dioxol-5-yl)-N-ethylpropan-2-amine	-	-
**a15**	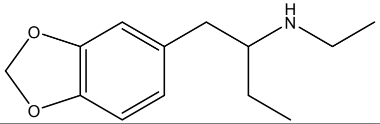	$C_{13}H_{19}NO_2$	167394-39-0	1-(benzo[d][1,3]dioxol-5-yl)-N-ethylbutan-2-amine	-	-

**a16**	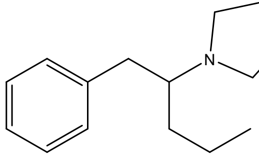	$C_{15}H_{23}N$	493-92-5	1-(1-phenylpentan-2-yl)pyrrolidine	-	-
**a17**	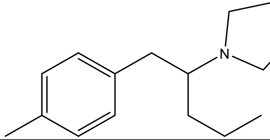	$C_{16}H_{25}N$		1-(1-(p-tolyl)pentan-2-yl)pyrrolidine	-	-
**a18**	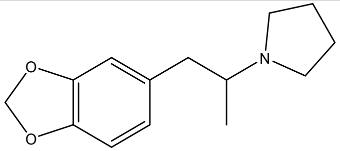	$C_{14}H_{19}NO_2$		1-(1-(benzo[d][1,3]dioxol-5-yl)propan-2-yl)pyrrolidine	-	-
**a19**	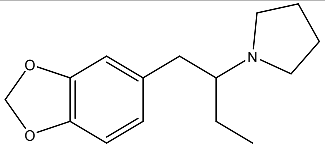	$C_{15}H_{21}NO_2$		1-(1-(benzo[d][1,3]dioxol-5-yl)butan-2-yl)pyrrolidine	-	-
**a20**	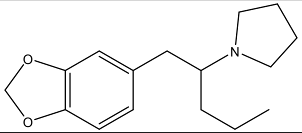	$C_{16}H_{23}NO_2$		1-(1-(benzo[d][1,3]dioxol-5-yl)pentan-2-yl)pyrrolidine	-	-
**a21**	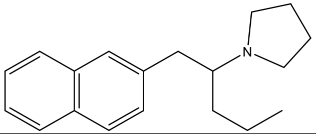	$C_{19}H_{25}N$		1-(1-(naphthalen-2-yl)pentan-2-yl)pyrrolidine	-	-
c01	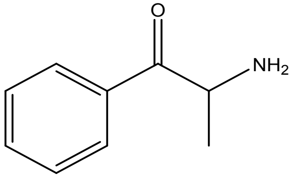	$C_9H_{11}NO$	71031-15-7	2-amino-1-phenylpropan-1-one	Cathinone	Benzoylethanamine or β-keto-amphetamine

**c02**	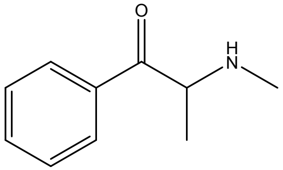	$C_{10}H_{13}NO$	5650-44-2	2-(methylamino)-1-phenylpropan-1-one	Methcathinone	Ephedrone, "cat", "jeff", "kat" or "intash"
**c03**	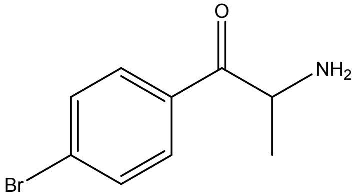	$C_9H_{10}BrNO$	408350-88-9	2-amino-1-(4-bromophenyl)propan-1-one	2-Amino-1-(4-bromophenyl)-1-propanone	-
**c04**	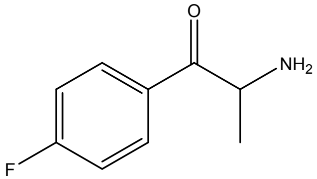	$C_9H_{10}FNO$	80096-51-1	2-amino-1-(4-fluorophenyl)propan-1-one	2-Amino-1-(4-fluorophenyl)-1-propanone	-
**c05**	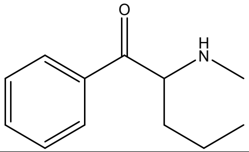	$C_{12}H_{17}NO$	879722-57-3	2-(methylamino)-1-phenylpentan-1-one	Pentedrone	α-methylaminovalerophenone
**c06**	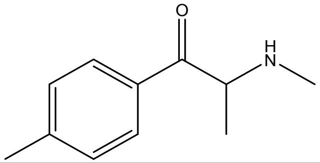	$C_{11}H_{15}NO$	1189805-46-6	2-(methylamino)-1-(p-tolyl)propan-1-one	Mephedrone	4-methylmethcathinone, 4-MMC, or 4-methylephedrone
**c07**	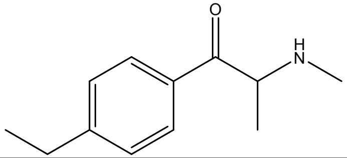	$C_{12}H_{17}NO$	1225622-14-9	1-(4-ethylphenyl)-2-(methylamino)propan-1-one	4-EMC	4-Ethylmethcathinone

**c08**	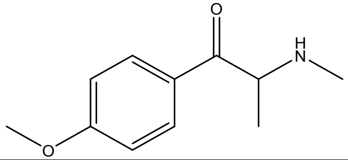	$C_{11}H_{15}NO_2$	530-54-1	1-(4-methoxyphenyl)-2-(methylamino)propan-1-one	Methedrone	Methoxyphedrine, bk-PMMA, PMMC, methoxyphedrine or 4-MeOMC
**c09**	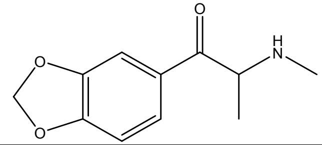	$C_{11}H_{13}NO_3$	186028-79-5	1-(benzo[d][1,3]dioxol-5-yl)-2-(methylamino)propan-1-one	Methylone	3,4-methylenedioxy-N-methylcathinone or MDMC
**c10**	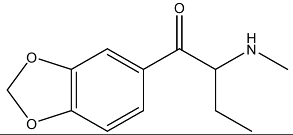	$C_{12}H_{15}NO_3$	17762-90-2	1-(benzo[d][1,3]dioxol-5-yl)-2-(methylamino)butan-1-one	Butylone	β-keto-N-methylbenzodioxolylbutanamine or βk-MBDB
**c11**	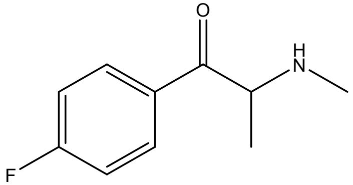	$C_{10}H_{12}FNO$	447-40-5	1-(4-fluorophenyl)-2-(methylamino)propan-1-one	4-FMC	Flephedrone or 4-fluoromethcathinone
**c12**	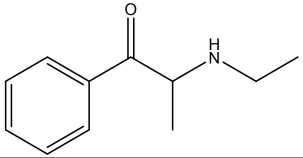	$C_{11}H_{15}NO$	18259-37-5	2-(ethylamino)-1-phenylpropan-1-one	ethcathinone	ethylpropion or ETH-CAT
**c13**	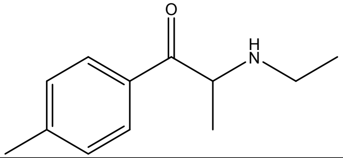	$C_{12}H_{17}NO$	1225617-18-4	2-(ethylamino)-1-(p-tolyl)propan-1-one	4-MEC	4-Methylethcathinone
**c14**	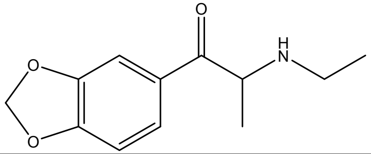	$C_{12}H_{15}NO_3$	1112937-64-0	1-(benzo[d][1,3]dioxol-5-yl)-2-(ethylamino)propan-1-one	Ethylone	MDEC or βk-MDEA

**c15**	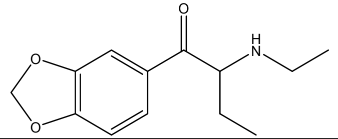	$C_{13}H_{17}NO_3$	802855-66-9	1-(benzo[d][1,3]dioxol-5-yl)-2-(ethylamino)butan-1-one	Eutilone	β-keto-1,3-benzodioxolyl-N-ethylbutanamine, bk-EBDB, or N-ethylbutylone
**c16**	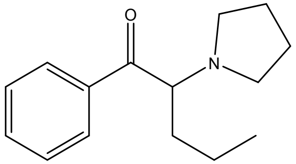	$C_{15}H_{21}NO$	14530-33-7	1-phenyl-2-(pyrrolidin-1-yl)pentan-1-one	α-PVP	α-Pyrrolidinopentiophenone, O-2387, β-keto-prolintane, prolintanone, or desmethylpyrovalerone
**c17**	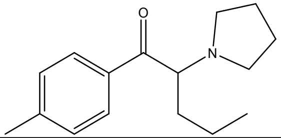	$C_{16}H_{23}NO$	3563-49-3	2-(pyrrolidin-1-yl)-1-(p-tolyl)pentan-1-one	pyrovalerone	Centroton, 4-Methyl-β-keto-prolintane, Thymergix or O-2371
**c18**	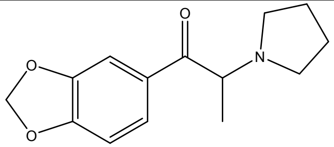	$C_{14}H_{17}NO_3$	783241-66-7	1-(benzo[d][1,3]dioxol-5-yl)-2-(pyrrolidin-1-yl)propan-1-one	MDPPP	3',4'-Methylenedioxy-α-pyrrolidinopropiophenone
**c19**	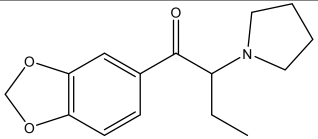	$C_{15}H_{19}NO_3$	784985-33-7	1-(benzo[d][1,3]dioxol-5-yl)-2-(pyrrolidin-1-yl)butan-1-one	MDPBP	3',4'-Methylenedioxy-α-pyrrolidinobutyrophenone
**c20**	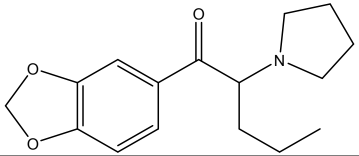	$C_{16}H_{21}NO_3$	687603-66-3	1-(benzo[d][1,3]dioxol-5-yl)-2-(pyrrolidin-1-yl)pentan-1-one	MDPV	Methylenedioxypyrovaleron e
**c21**	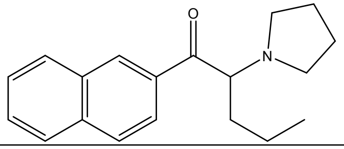	$C_{19}H_{23}NO$	850352-53-3	1-(naphthalen-2-yl)-2-(pyrrolidin-1-yl)pentan-1-one	Nafirona	O-2482 or naphthylpyrovalerone

Table S2. Summary of bond lengths (Å), bond angles (°), and dihedrals (°) for the computationally and experimentally obtained structures (Exp.).

**Bond lengths /Å**							
**Bonds**	**Exp. 1**	**Exp. 2**	**Exp. 3**	**Exp. 4**	**B3LYP ‡**	**B3LYP †**	**MP2**
C1-C2	1.51	1.51	1.52	1.48	1.49	1.50	1.50
C2-C3	1.52	1.54	1.54	1.50	1.55	1.53	1.54
C3-C4	1.51	1.51	1.52	1.49	1.53	1.55	1.53
C3-N5	1.51	1.47	1.48	1.51	1.49	1.46	1.47
C3-H6	1.04	1.00	1.00	1.05	1.12	1.09	1.10
C2-O12	1.42	1.43	1.43	-	1.21	1.22	1.22
N5-H7	1.03	0.88	0.87	0.89	1.02	1.01	1.02
C16-C17	1.37	1.37	1.39	1.33	1.40	1.39	1.40
C1-C14	1.38	1.39	1.39	1.34	1.40	1.40	1.40
C1-C13	1.38	1.39	1.40	1.33	1.40	1.40	1.40
C13-H18	0.93	0.96	0.95	0.91	1.09	1.08	1.08
C4-H9	0.98	0.97	0.98	0.96	1.10	1.09	1.09
**Bond angle /°**							
**Angles**	**Exp. 1**	**Exp. 2**	**Exp. 3**	**Exp. 4**	**B3LYP ‡**	**B3LYP †**	**MP2**
C1-C2-C3	110.63	113.47	111.53	114.75	116.24	119.84	119.67
C2-C3-C4	113.82	113.22	112.72	113.65	109.66	108.13	111.12
C2-C3-N5	107.18	113.82	108.35	108.85	106.04	108.68	107.97
C2-C3-H6	107.31	103.96	109.02	114.34	109.56	109.04	107.06
C1-C2-O12	114.03	108.86	111.95	-	122.53	120.54	120.65
C2-C1-C13	121.71	122.10	120.87	121.95	119.03	118.04	117.57
C17-C16-C15	120.28	119.76	119.94	119.53	120.15	119.86	119.98
C17-C16-H20	123.04	119.93	120.03	120.22	119.91	120.11	120.02
H8-N5-H7	109.65	100.27	105.65	109.47	106.90	108.98	106.13
C4-C3-N5	109.98	109.29	108.59	106.66	114.63	114.51	114.87
O12-C2-C3	105.25	109.41	107.44	-	121.23	119.52	119.68
**Dihedral /°**							
**Diedral**	**Exp. 1**	**Exp. 2**	**Exp. 3**	**Exp. 4**	**B3LYP ‡**	**B3LYP †**	**MP2**
C1-C2-C3-C4	-62.96	62.00	-56.45	-63.75	142.19	79.35	164.23
C1-C2-C3-N5	175.21	-63.61	-175.65	177.56	-93.53	-155.79	-68.95
C1-C2-C3-H6	58.84	-179.52	64.79	55.89	22.32	-37.99	45.29
O12-C2-C3-C4	60.65	-176.22	-179.49	-	-36.77	-97.09	-14.79
O12-C2-C3-N5	-61.17	58.17	60.32	-	87.51	27.77	112.03
O12-C2-C3-H6	-177.55	-57.75	-58.24	-	-156.64	145.57	-133.73
C17-C13-C1-C2	177.33	176.95	176.42	177.69	-179.73	179.83	178.54
C15-C16-C17-C13	0.91	-0.60	0.85	2.21	0.05	0.05	0.18
C15-C16-C17-H19	-175.43	-179.57	-179.17	-177.77	-179.48	-179.94	-179.87
C13-C1-C2-O12	160.83	-40.65	42.18	-	41.13	9.65	9.08

‡ - using the 6-31G** basis; † - using the TZVP basis; - : indicate data not available in the reference article used.

Table S3. HOMO−LUMO difference of amphetamines and the reaction product with FITC.

Amphetamine	GAP/eV	GAP/ kcal mol^{-1}	Amphetamine + FITC	GAP/eV	GAP/ kcal mol^{-1}
a01	6.375	147.00	a01+FITC	3.225	74.36
a02	6.016	138.73	a02+FITC	3.203	73.87
a04	6.138	141.55	a04+FITC	3.193	73.63
a05	6.066	139.88	a05+FITC	3.202	73.83
a06	6.077	140.13	a06+FITC	3.092	71.30
a07	5.680	130.98	a07+FITC	3.125	72.07
a08	5.523	127.37	a08+FITC	3.287	75.81
a09	5.514	127.17	a09+FITC	3.145	72.54
a10	5.899	136.02	a10+FITC	3.150	72.63
a11	5.522	127.33	a11+FITC	3.184	73.43
a12	5.513	127.14	a12+FITC	3.353	77.31
a13	6.375	147.00	a13+FITC	3.204	73.89
a14	6.016	138.73	a14+FITC	3.098	71.45
